# Exploring the Use of Persuasive System Design Principles to Enhance Medication Incident Reporting and Learning Systems: Scoping Reviews and Persuasive Design Assessment

**DOI:** 10.2196/41557

**Published:** 2024-03-21

**Authors:** Kiemute Oyibo, Paola A Gonzalez, Sarah Ejaz, Tasneem Naheyan, Carla Beaton, Denis O’Donnell, James R Barker

**Affiliations:** 1 Department of Electrical Engineering and Computer Science, Lassonde Research Centre York University North York, ON Canada; 2 Faculty of Management Dalhousie University Halifax, NS Canada; 3 Pharmapod Think Research Corporation Toronto, ON Canada; 4 CareRx Clinical Excellence Toronto, ON Canada

**Keywords:** medication incident, reporting system, persuasive technology, persuasive design, medication, persuasive system design, pharmacy, pharmaceutic, pharmacology, drug reporting, drug event, adverse event, incident management

## Abstract

**Background:**

Medication incidents (MIs) causing harm to patients have far-reaching consequences for patients, pharmacists, public health, business practice, and governance policy. Medication Incident Reporting and Learning Systems (MIRLS) have been implemented to mitigate such incidents and promote continuous quality improvement in community pharmacies in Canada. They aim to collect and analyze MIs for the implementation of incident preventive strategies to increase safety in community pharmacy practice. However, this goal remains inhibited owing to the persistent barriers that pharmacies face when using these systems.

**Objective:**

This study aims to investigate the harms caused by medication incidents and technological barriers to reporting and identify opportunities to incorporate persuasive design strategies in MIRLS to motivate reporting.

**Methods:**

We conducted 2 scoping reviews to provide insights on the relationship between medication errors and patient harm and the information system–based barriers militating against reporting. Seven databases were searched in each scoping review, including PubMed, Public Health Database, ProQuest, Scopus, ACM Library, Global Health, and Google Scholar. Next, we analyzed one of the most widely used MIRLS in Canada using the Persuasive System Design (PSD) taxonomy—a framework for analyzing, designing, and evaluating persuasive systems. This framework applies behavioral theories from social psychology in the design of technology-based systems to motivate behavior change. Independent assessors familiar with MIRLS reported the degree of persuasion built into the system using the 4 categories of PSD strategies: primary task, dialogue, social, and credibility support.

**Results:**

Overall, 17 articles were included in the first scoping review, and 1 article was included in the second scoping review. In the first review, significant or serious harm was the most frequent harm (11/17, 65%), followed by death or fatal harm (7/17, 41%). In the second review, the authors found that iterative design could improve the usability of an MIRLS; however, data security and validation of reports remained an issue to be addressed. Regarding the MIRLS that we assessed, participants considered most of the primary task, dialogue, and credibility support strategies in the PSD taxonomy as important and useful; however, they were not comfortable with some of the social strategies such as cooperation. We found that the assessed system supported a number of persuasive strategies from the PSD taxonomy; however, we identified additional strategies such as tunneling, simulation, suggestion, praise, reward, reminder, authority, and verifiability that could further enhance the perceived persuasiveness and value of the system.

**Conclusions:**

MIRLS, equipped with persuasive features, can become powerful motivational tools to promote safer medication practices in community pharmacies. They have the potential to highlight the value of MI reporting and increase the readiness of pharmacists to report incidents. The proposed persuasive design guidelines can help system developers and community pharmacy managers realize more effective MIRLS.

## Introduction

### Overview

Medication errors are one of the leading causes of death in many countries worldwide [1,2]. For example, in the United States alone, 7000 to 9000 patients die annually owing to these errors. In Canada, where medical errors (labeled as the third leading cause of death after cancer and heart disease) account for 28,000 deaths annually, every minute and 18 seconds a patient gets harmed because of unintended errors, with medication errors being the most frequent [3]. Wrong medication (eg, because of similar naming, similar packaging, illegible handwriting, and incorrect drug selection) and wrong dose are among the most common medication errors in community pharmacies [3-5]. In particular, advanced drug preparation and administration without double checking [6] and heavy workflow [7] have been identified as key contributing factors to medication errors. However, there may be many more contributing underlying factors that go unreported by pharmacists and other health professionals. For example, a survey on medication administration errors among nurses in South Korea showed that 63.6% of the respondents had been involved in medication errors once or more in the previous month. However, only 28.3% of the participants reported the incidents [6]. Underreporting of medication errors, which is a global issue [8-11], has several implications bordering on shared learning, patient safety, and financial cost. In the United States, for example, psychological or physical pain and distress aside, “the total cost of looking after patients with medication-associated errors exceeds US $40 billion each year, with over 7 million patients affected” [4]. Moreover, underreporting of medication errors and incidents might limit individual and organizational learning from their occurrence [12,13].

The continuous evolution of pharmacotherapy and changing demands on the community pharmacy necessitate constant vigilance to detect new types of medication errors [14]. In a study among hospital pharmacists in South Korea, Hee-Jin et al [15] found that “five or more near misses per month were experienced by 14.8%, 4.3%, and 43.9% of respondents for dispensing, administration, and prescribing errors, respectively.” Moreover, research has shown that medication errors that lead to patient harm are common in medical care including community pharmacy [2,16-19]. Frequent reporting of all medication incidents (MIs) and near-miss events has the potential to improve patient safety through shared learning, which will enable the reduction of recurrence and prevention of MIs in the future [20,21]. Without adequate user reporting, none of the laudable objectives of reporting systems, including identification of gaps and resource development to support patient safety, can be achieved [7]. Medication error reporting is a common metric used to assess the quality of care provided by the health care system [21]. However, research has shown that employees are less motivated to report medication errors [22-25]. Hence, there is a need to find ways to motivate pharmacists and pharmacy technicians to report MIs more often to foster shared learning, prevention of recurrence, and patient safety. The question then is, *How can we motivate pharmacists and pharmacy technicians to report MIs more frequently using persuasive design principles embedded in digital technologies?*

**Figure 1:**
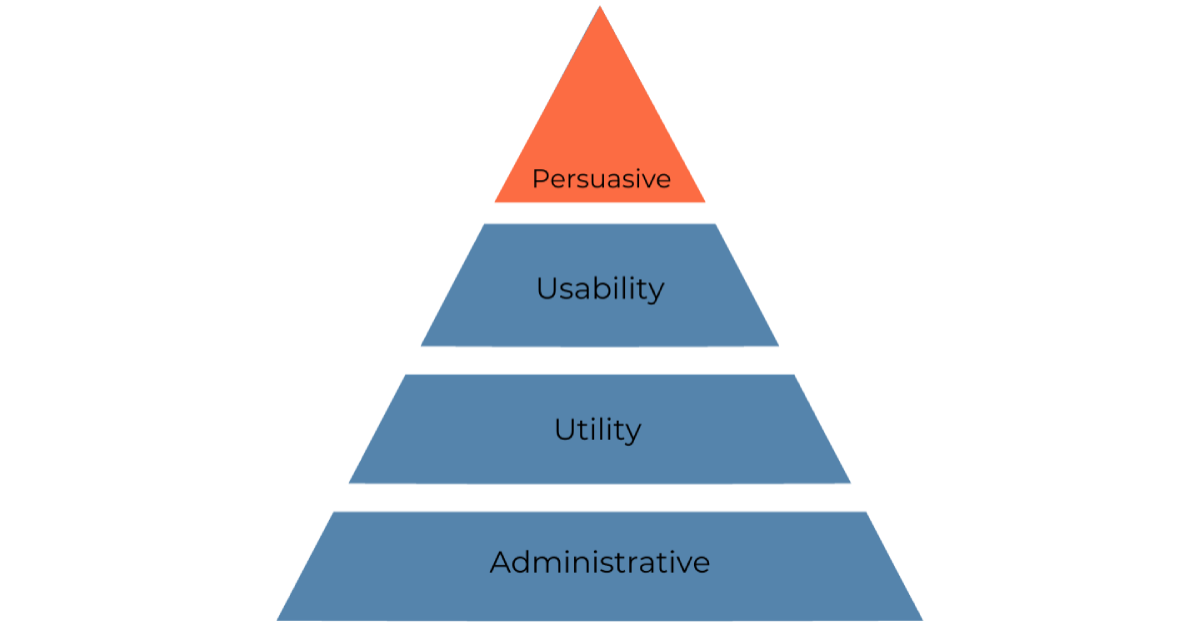
Four key design principles for Medication Incident Reporting and Learning Systems in community pharmacy.

Although some guiding principles have been proposed to alleviate the barriers to MI reporting, these principles, from a user experience (UX) design perspective, are not aimed at motivating pharmacists to report MIs regularly. From our literature search, we identified 4 categories of principles that can guide the design of Medication Incident Reporting and Learning Systems (MIRLS) to improve their adoption and usability. They include administrative principles, usability, utility principles, and persuasive design principles (Figure 1). Administrative principles refer to the organizational processes and policies implemented to enable and encourage employees to report medication errors regularly without fear of consequences. These principles form the basis of MIRLS, upon which the other categories of principles build. Usability and utility principles refer to the UX features that enable a user to report medication errors with ease, effectiveness, efficiency, and satisfaction [26]. Persuasive principles refer to the motivational affordances of a system that facilitate, nudge, and motivate a user to report medication errors. Current MIRLS mainly focus on the administrative, usability and utility-based principles. Typical examples of administrative principles include voluntary use, anonymity, confidentiality, and nonpunitive consequences. Examples of usability and utility-based principles, particularly in the Think Research and Pharmapod system, include ease of use, use of a standard taxonomy, searchability, retrievability, report generation, and root cause analysis [7,14,27].

Apart from the administrative, usability, and utility principles, we argue that persuasive design principles hold potential to increase MI reporting among pharmacists. Persuasive design principles embedded in digital technologies, also known as persuasive technology, can motivate increased reporting of MIs from community pharmacies, as research in other health domains has shown [28]. Hence, this study proposes the use of persuasive design principles, which build on the 3 other categories of principles (Figure 1), to motivate users of MIRLS to report incidents and near misses more often.

Using Think Research, also known as Pharmapod, a cloud-based MIRLS for reporting and reducing incidents in community pharmacies [29], as a case study, this study (1) assesses 1 MIRLS based on the Persuasive System Design (PSD) taxonomy proposed by Oinas-Kukkonen and Harjumaa [30] and (2) proposes persuasive design guidelines to help community pharmacy stakeholders at multiple levels (eg, facility, provincial, and national) integrate persuasive features into their MIRLS. The PSD taxonomy is a widely used framework in the persuasive technology domain for analyzing, designing, implementing, and evaluating persuasive systems. Persuasive strategies from the PSD taxonomy can enhance MIRLS, making them more effective in promoting patient safety and shared learnings among practitioners [31,32]. Moreover, the study presents a summary of the results on the relationship between medication errors and harm and the information system–based barriers to MI reporting to ground the research.

### Background and Related Work

In this section, we present an overview of relevant studies on the relationship between medication errors and patient harm and the organizational and information system barriers to reporting.

#### Medication Errors and Patient Harm

Several studies have been conducted to investigate the prevalence, nature, severity, and effects of MIs. West et al [16] investigated the relationship between medical errors and patient harm in primary care. They found that clinical harm to patients was reported in >10% of the 608 primary care medical error reports, with prescription-related errors most frequently linked to clinical harm. Similarly, Robb et al [17] investigated the relationship between medication and patient harm in hospitals in New Zealand. The authors confirmed the findings of earlier studies that showed that medication-related harms were common in both hospitals and the community, posing a substantial burden for patients and the health care system. In particular, they found that 923 harms were identified among 751 patients, with 28% of them experiencing ≥1 of the medication-related harms. They also found that older and female patients and those who had an increased length of stay were more likely to be harmed. Moreover, 65% of the harms occurred during an inpatient stay and 29% originated from the community and resulted in an admission. Riordan et al [18] investigated discharge prescription errors and their propagation after the discharge of patients. They found that 43% of the patients included in the study experienced postdischarge medication errors, with 86% of them being at risk of moderate harm. Moreover, 88% of the errors were discharge prescription errors that persisted after the discharge.

Most recently, Alqenae et al [2] conducted a systematic review, which they regarded as the first, to explore the prevalence and nature of medication errors and adverse drug events after hospital discharge. The review found that the median rate of medication error was approximately 50% among adult and older patients after hospital discharge, with approximately 20% of the patients in the studies reported to be affected by adverse drug events (such as antibiotics, antidiabetics, analgesics, and cardiovascular drugs) after hospital discharge. Panagioti et al [19] conducted a systematic review and meta-analysis of the prevalence, severity, and nature of preventable patient harm across a range of medical care settings. They found that 5% of the patients were exposed to preventable harm in medical care and 25% of the incidents, which are drug related, accounted for the largest proportion of preventable patient harm, with 12% of the preventable patient harms being severe or leading to death. They asserted that there are limited quality improvement practices specifically targeting incidents that cause preventable harm to patients. They added that designing and implementing evidence-based mitigation strategies specifically targeting preventable patient harm could lead to substantial service quality improvements that are cost effective. This conclusion by Panagioti et al [19], coupled with the prevalence of medication errors in community pharmacy, partly informs this conceptual paper aimed to incorporate persuasive principles in MIRLS to increase medication error reporting and patient safety.

#### Organizational Barriers to MI Reporting

Researchers have identified several organizational barriers (both administrative and personal) leading to underreporting of medication errors and incidents in community pharmacy [21]. In the long run, these barriers can adversely affect patient safety owing to lack of shared learning among pharmacists within and across organizations because of underreporting [12,13]. Key barriers include fear of consequences such as punitive and disciplinary actions, negative or lack of administrative feedback, poor work climate or culture, inadequate training, and time constraint (Textbox 1) [7,8,21]. For example, Bahadori et al [9] found that the most important reasons for not reporting medication errors were administrative factors including the process of reporting and fear of the consequences of reporting. Research has also shown that personal (ie, sociodemographic) factors can impact medication error reporting. For instance, Aljabari and Kadhim [8] found that younger and lesser experienced professionals and staff with shorter employment periods were less likely to report medication errors. We argue that administrative barriers (such as time constraint and high workload) and perceived low value of the reporting system could be mitigated by using persuasive technologies to facilitate and ensure convenient reporting of MIs and errors. For example, persuasive design features (such as reminders to complete saved draft reports, notifications about the utility and value of reporting, and encouraging messages) may facilitate MI reporting.

**Administrative barriers to reporting medication errors and incidents.**

**Fear of consequences**

- Negative consequences such as blame, shame, professional reputation damage, relationship damage, loss of privileges, medical malpractice lawsuit, relief from certain duties, and loss of job [4,9,33,34].

**Lack of feedback**

- Lack of useful feedback or negative feedback from administrative teams, such as pharmacy managers, regarding previously reported medication errors [33,34].

**Poor work climate or culture**

- Blaming staff and not the system or culture, poor support system, poor teamwork, poor organizational leadership, and lack of confidentiality in handling reports [33,35].

**Miscommunication**

- Poor communication among staff or between staff and patients [36].

**Inadequate training of staff**

- Difficulty in using the reporting system, poor understanding of the importance or value of reporting, poor understanding of errors, lack of clear definition of incident or near miss, and lack of a well-defined protocol on what events need to be reported [21,35].

**Time constraint**

- Work pressure and the lack of budgeted time to properly report errors, especially in the midst of a busy work schedule and high workload resulting in lack of enough breaks [7,35,36].

#### Information System Barriers to Patient Safety

Research has identified technological barriers that hamper patient safety in different health information systems and domains [37-40]. The primary barrier among them is the usability and poor design of health information systems [41]. Ratwani et al [42] found across 3 health care institutions that the usability of electronic health records accounted for more than a third of medication errors in 9000 pediatric patient safety reports. Kushniruk et al [43] evaluated the usability of a handheld prescription writing application. They found various usability problems (most of which relate to interface design) and actual errors in entering prescription data. In particular, they found that certain types of usability problems such as display visibility and ergonomics-related wrong data entry were closely linked to the occurrence of specific types of errors in medication prescription. More recently, Adams et al [37] investigated the medication errors associated with health information technology use and the harm caused to the patient. They found that 55.85% (1508/2700) of the manually reviewed reports described a medication error associated with information technology use and 49.7% (750/1508) of these caused harm to the patient. In particular, they found that 97.35% (1468/1508) of the medication errors associated with information technology were related to usability issues including data entry, workflow support, and alerting. On the basis of these findings, in the current MIRLS domain, we set out to uncover the information technology barriers that border on the usability and utility principles (Figure 1), which may lead to the low perceived value, utility, and use of MIRLS.

#### PSD of MIRLS

PSD was pioneered by Fogg [44] in the early 2000s in his seminal book, “Persuasive technology: using computers to change what we think and do.” This entails the application of behavioral theories from social psychology in the design of technology-based systems to motivate behavior change. Hence, persuasive technology is defined as a motivational tool intentionally designed to change human attitudes and behaviors through persuasive techniques grounded in social psychology [44]. Fogg [44] first proposed a set of 7 persuasive strategies to motivate behavior change. Subsequently, Oinas-Kukkonen and Harjumaa [30] extended the list to 28 persuasive strategies, which are categorized into 4 functional groups (primary task support, dialogue support, social support, and system credibility support), each comprising 7 persuasive strategies. Oyibo [45] extended the primary task support and dialogue support groups with goal setting and verbal persuasion, respectively, increasing the total number of strategies in the PSD taxonomy to 30. The primary task support category, which includes tunneling, tailoring, and self-monitoring, is aimed at helping the user to perform a target behavior easily and effectively. The dialogue support category, which includes praise, reward, and suggestion, is aimed at motivating the user to perform the target behavior through feedback and dialogue with the persuasive system. The social support category, which includes social learning, social comparison, and competition, is aimed at motivating the user through social influence to perform the target behavior. Finally, the system credibility support category, which includes trustworthiness, surface credibility, and authority, is aimed at increasing the user’s trust in the system by making the system look professional and credible [46].

Incorporating persuasive features into MIRLS has the potential to improve the rate of error reporting. St-Maurice et al [28] showed that, on average, the percentage of same-day data entries can be increased by 10% for each user by introducing new persuasive design features into a data entry system. On the basis of this prior research finding, we propose guidelines for incorporating persuasive design principles, drawn from the PSD taxonomy, into MIRLS using the Think Research or Pharmapod Incident Management (IM) system as a case study. The PSD taxonomy, which comprises 4 categories of persuasive strategies (primary task support, dialogue support, social support, and system credibility support), is a framework for analyzing, designing, implementing, and evaluating persuasive systems. A systematic review by Win et al [47] showed that primary task support and dialogue support are the most commonly used categories of persuasive strategies in medication management information systems. The review reported that tailoring, self-monitoring, and reminders, which belong to the primary task support category, are more likely to be implemented in medication management information systems than other persuasive strategies. In the case of MIRLS, the proposed persuasive strategy guidelines are aimed at enhancing system utility and facilitating the reporting of near misses and incidents. Research shows that the higher the perceived usefulness of health systems, the higher the number of users who find them more persuasive [48].

## Methods

### Overview

A total of 2 types of methods were used to address 3 research questions (RQs). They include scoping review and assessment of an existing MIRLS based on administrative, usability, utility, and persuasive features. The RQs are as follows:

1. RQ1. Is there a relationship between medication errors and patient harm?
2. RQ2. What are the information system–based barriers preventing pharmacists and pharmacy technicians from reporting medication errors?
3. RQ3. How can we motivate them to report MIs more frequently using persuasive design principles embedded in digital technologies?

### Ethical Considerations

The assessment of our target system was aimed at quality improvement, thus ethical approval was not required [49,50]. 

### Scoping Reviews

To address the first 2 RQs, the authors (KO, SE, and TN) conducted 2 scoping views in August 2023. The first review investigated the relationship between medication errors and patient harm in the pharmacy domain. The second review aimed to uncover usability and utility-related barriers to medication error reporting. We retrieved articles from 6 databases for each study, screened the articles, extracted the relevant data, and presented the results. For the first review, a total of 820 articles were retrieved from PubMed (n=41), Public Health Database (n=89), ProQuest (n=451), Scopus (n=97), ACM (n=42), and Global Health (n=22) using the search string: “(Medic* OR prescri* OR administ* OR drug*) AND (error* OR incident* OR accident* OR nearmiss* OR ‘near miss*’ OR mistake*) AND patient AND (harm* OR hurt* OR injur* OR wound* OR bruise* OR impairment* OR afflict*) AND pharmac*.” A total of 215 duplicates were removed to arrive at 605 unique articles. These articles were screened based on title or abstract to arrive at 91 articles. Next, a full-text review was conducted to arrive at 14 included articles after excluding 77 ineligible articles. Finally, 3 more articles were included to the 14 through Google Scholar search, resulting in 17 articles for the final data analysis. For the second review, a total of 849 articles were retrieved from PubMed (n=268), Public Health Database (n=44), ProQuest (n=90), Scopus (n=448), ACM (n=10), and Global Health (n=45) using the search string: “(Medic* OR prescri* OR administ* OR drug*) AND (error* OR incident* OR accident* OR nearmiss* OR ‘near miss*’ OR mistake*) AND (report* OR submi* OR log*) AND (system* OR application* OR website* OR tool* OR platform* OR interface* OR technolog*) AND pharmac* AND (barrier* OR hinderance* OR obstacle* OR drawback* OR setback* OR deterrent* OR limitation* OR shortcoming*.” A total of 303 duplicates were removed to arrive at 546 unique articles. These articles were screened based on title or abstract to arrive at 12 articles. Upon the full-text review, we arrived at zero article for data extraction and analysis. Moreover, based on Google Scholar search, we found 1 article [13] that investigated the usability of MIRLS called the Medication Error Reporting App. However, this study did not investigate the relationship between the usability of the app and medication error.

### Overview and Initial Assessment of an Existing MIRLS

The authors (KO and PAG) analyzed the Think Research or Pharmapod MIRLS, which is a cloud-based software platform for reporting medication errors (incidents and near misses). As stated on its website, Think Research or Pharmapod describes itself as “the first platform of its kind to pool and share patient safety data across borders, monitoring trends and causes behind medication errors, and empowering healthcare professionals locally to improve their practice” [29]. Our initial review of the system assessed it against the 3 key design principles shown in Figure 1. To assess the administrative and usability and utility principles, the first 2 authors went through the Think Research or Pharmapod system from one interface to another to elicit the supported principles. Next, we used the PSD taxonomy as an assessment framework and 3 assessors (study participants) to identify persuasive strategies fully or partially implemented in the Think Research or Pharmapod IM system. We first assessed the system to identify the existing persuasive strategies and then gathered data from 3 experienced users to propose opportunities for improvement. One of the authors, the vice president of the Quality Improvement and Innovations of Think Research or Pharmapod, arranged for 3 independent and experienced users of the Think Research or Pharmapod IM system from different pharmacies to assess the system against the PSD taxonomy and items. The first assessor was a pharmacist who had 1.5 years of experience using the system. The second assessor was a director in a health care company focused on patient and staff safety, with 1 year of experience working with the system. The third assessor was a senior technology manager at a leading Canadian pharmacy company, with 4 years of experience working with the system.

The authors (KO and PAG) asked the assessors to independently indicate whether each persuasive strategy in the PSD taxonomy is important or useful, present in the system or not, and where it could be found in the system. The implementation of each strategy from the PSD taxonomy was described to the participants in a tabular form. The participants independently responded to the questions and then came together to discuss and confirm their responses and resolve their differences with the first 2 authors. If at least 2 of the 3 assessors indicated or agreed that a given persuasive strategy is important and useful, “yes” is entered into the associated cell in the table, otherwise, “no.” Similarly, if at least 2 assessors agreed that the strategy was present in the system (ie, said “yes”), “√” is entered into the cell associated with the status column. However, if ≥2 assessors agreed that the strategy was not present in the system (ie, said “no”), “X” is entered into the associated cell under the status column. Moreover, if at least 1 of the assessors agreed that the strategy was present in the system, but the implementation was limited, “√*” standing for “present but could be improved” is entered into the associated cell under the status column.

## Results

In this section, we present the results of the scoping reviews and the initial assessment of the Think Research or Pharmapod IM system.

### Scoping Reviews

#### Medication Errors and Patient Harm

In the first review, 41% (7/17) of the included articles originated from North America (United States [16,51-53], Canada [54,55], and Mexico [56]), 29% (5/17) from Europe (United Kingdom [12], Ireland [18], the Netherlands [57], Sweden [58], and Spain [59]), 23% (4/17) from Asia (Saudi Arabia [60,61], China [62], and Korea [63]), and 6% (1/17) from Oceania (New Zealand [17]). The articles were published between 2001 and 2023, with most of the articles (3/17, 18%) published in 2023. Most of the target populations were from North America (7/17, 41%), followed by Asia (5/17, 29%), Europe (4/17, 23%), and Oceania (1/17, 6%). Of the 17 articles, 1 (6%) each focused on target populations in Africa and South America. Table 1 shows 16 types of harms elicited from the included articles. These were caused by 59 types of medication errors such as wrong drugs, missing or wrong patient weight, prescription errors, dosing error, wrong or unclear dose or strength, wrong patient, and wrong duration, each of which was reported by at least 2 articles. Significant or serious harm was the most frequent harm; it was reported by 65% (11/17) of the articles, followed by death or fatal harm (7/17, 41%) and no harm or potential harm (4/17, 23%).

**Table 1:** Type or severity of harm caused by medication errors and the number of articles associated with them (N=17).

Type or severity of harm	Articles, n (%)
Significant or serious harm	11 (65)
Death or fatal harm	7 (41)
No harm or potential harm	4 (23)
Inconvenience	3 (18)
Adverse drug events	3 (18)
Mild harm	2 (12)
Moderate harm	2 (12)
Temporary injury or harm	2 (12)
Prolonged hospitalization	2 (12)
Life-threatening harm	2 (12)
Nonlife threatening	1 (6)
Risk to patient or others	1 (6)
Unstable situation	1 (6)
Unknown harm	1 (6)
Permanent harm	1 (6)
Intervention required	1 (6)

#### Information System Barriers to MI Reporting

One article [13] that investigated the usability of an MIRLS prototype called Medication Error Reporting App found that there was significant improvement in the mean usability score throughout the development process (*P*<.001). However, this mean improvement in usability did not impact the mean time to report medication errors using the app because the mean time was not significantly different between the phases of the development process. Overall, it was found that the testers including pharmacists found the app easy to use, but doctors and nurses were unfamiliar with the medication terms used, especially the medication process in which error occurred and type of error. More importantly, the authors reported that although testers might be willing to adopt the app to make reports in the future, they were apprehensive about data protection issues such as security and abuse of feedback featured in the app [13].

### Initial Assessment of Existing MIRLS

In this section, we present the results that emanated from the initial assessment of the Think Research or Pharmapod IM system based on administrative, usability, utility, and persuasive principles.

#### Administrative and Usability and Utility Principle Support

The assessed system supported at least 75% (6/8) of the administrative guiding principles shown in Textbox 2, including voluntariness, anonymity, confidentiality of information, and nonpunitive measures. It also supported all 7 usability and utility-based principles, including ease of use, searchability and retrievability, standard taxonomy, report generation, and root cause analysis (Table 1).

**Items and questions asked of assessors.**

**Strategy code**

- A codeword representing the persuasive strategy.

**System capability**

- A description of the persuasive strategy.

**Important or useful**

- An indication of the importance or usefulness of the strategy (yes or no).

**Present in system**

- An indication of the presence of the strategy in the system (yes or no).

**Interface, tab, or comment**

- Provision of the system interface or tab where the persuasive strategy can be found or a comment by the assessor.

**Figure 2:**
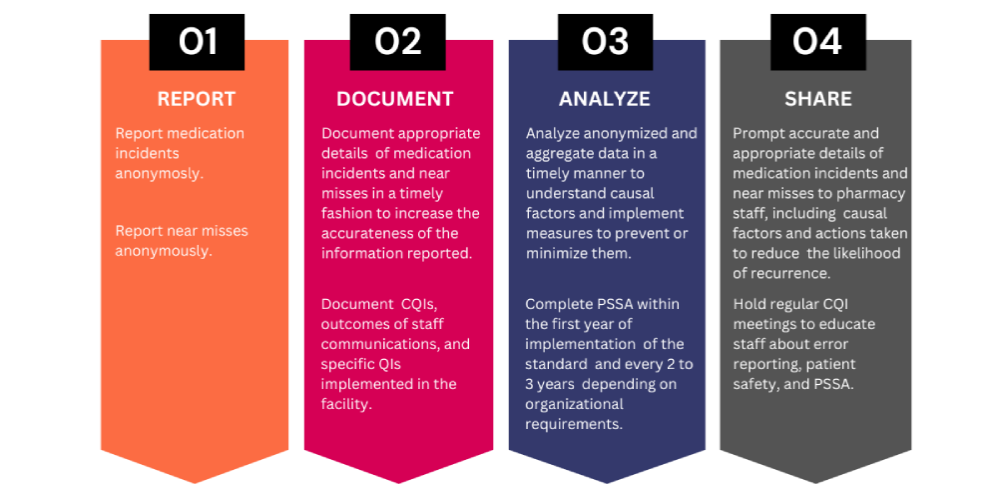
Four key elements of patient medication safety.

**Figure 3:**
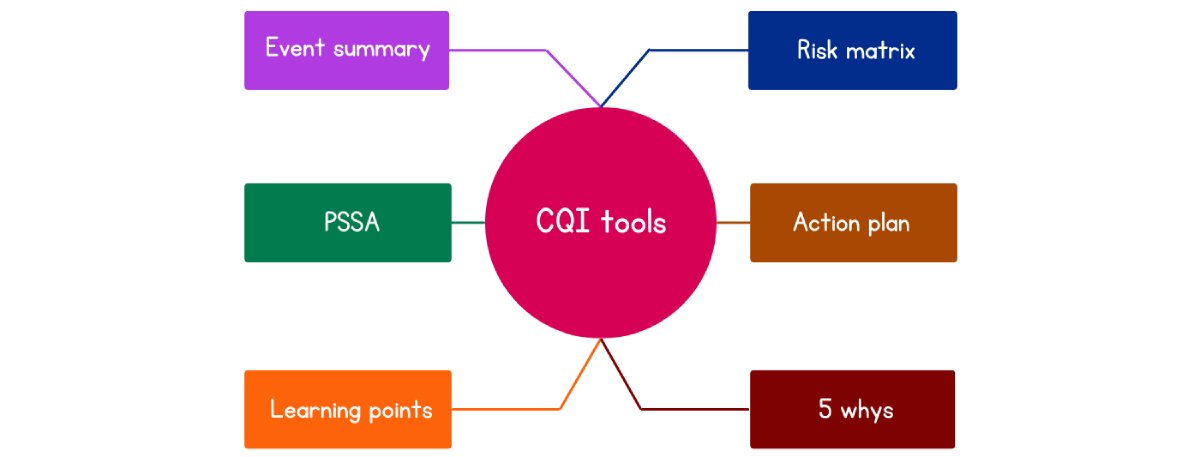
Six continuous quality improvement (CQI) tools in the studied system aimed at fostering fundamental change among pharmacy team members. PSSA: pharmacy safety self-assessment.

Moreover, the system promotes 4 key elements of patient medication safety: report, document, analyze, and share (Figure 2). The analyze and share elements are in addition supported by 6 main continuous quality improvement (CQI) tools. These tools are intended to foster patient safety in community pharmacy within a pharmacy team [20,64]. The tools include event summary, risk matrix, 5-whys template, action plan, learning points, and pharmacy safety self-assessment (Figure 3). An event summary is an incident and a root cause analysis tool. The risk matrix is a color-coded matrix that facilitates the assignment of a risk score based on the probability of recurrence of the incident or near miss at a specific severity level and its impact on a patient if it were to recur. The 5-whys is a tool that facilitates the analysis of an incident or near miss by answering the fundamental question, “Why did the incident occur?” 5 times. The 5-whys is a simple and well-recognized tool for determining the cause and effect of an incident objectively. Action plans is a tool to create and track smart actions of improvement. Learning points organizes identified gaps, for example, in workflows and processes and provides a means to share these learnings. Finally, the pharmacy safety self-assessment is a tool that allows the pharmacy team to proactively identify risks that may compromise patient safety and implement safe medication measures to address them [64].

#### Persuasive Principle Support

Tables 2-5 show the results of the assessment of the Think Research or Pharmapod IM system based on the primary, dialogue, social, and credibility support categories of the PSD taxonomy, respectively. The first column of Table 2 captures the coded name of the strategy and its description, the second column describes a yes or no response on the importance and usefulness of the strategy, and the third column describes a yes or no response on the presence of the strategy in the system (ie, status). A fourth column was also provided for the assessors to comment on the assessment of each strategy, for example, the location of the strategy in the system.

**Table 2:** Guidelines for incorporating the primary task support principles into Medication Incident Reporting and Learning Systems.

Strategy and implementation			I or U^a^		Status
**Reduction**					
	Break down the medication incident and near-miss reporting process into a few simple steps to facilitate reporting [65].	Yes		✓^b^	
**Tunneling**					
	Guide the user through the reporting process in a step-by-step fashion, just as a software installation wizard [47].	Yes		X^c^	
**Goal setting**					
	Allow the user to set a goal, for example, minimum number of errors or CQI^d^ reports to be submitted over a given period such as a week or month.	Yes		X	
**Self-monitoring**					
	Allow the user to track their progress after setting a report-based goal or when submitting a report, for example, through the display of a progress bar.	Yes		X	
	Allow the user to view their number of completed and uncompleted reports and averages per week, month, or year (eg, on their dashboard).	Yes		✓	
	Allow the user to track the levels of usefulness of their reports (eg, CQI, incident, or near miss) to others, for example, other users or colleagues “like” their anonymous reports as obtainable in YouTube and Facebook.	Yes		X	
**Tailoring**					
	Tailor what the user sees (eg, user profile, chart, content, and information) using group-based characteristics such as work experience and designation or role.	Yes		✓*^e^	
**Personalization**					
	Personalize the system (eg, information, report, and reminder) based on their interaction, for example, letting the user know where they left off or reminding them about incomplete tasks when they log in [7,66].	Yes		X	
**Customization**					
	Allow the user to customize the system (eg, profile, chart, content, information, and reminder) to suit their needs and preferences [66].	Yes		✓*	
**Simulation**					
	Show the user a cause-and-effect relationship of the benefit of incident or near miss or CQI reporting, for example, a study chart showing the higher the incidents reported, the lower the number of recurrences.	Yes		X	
**Rehearsal**					
	Provide a new user with a simulated environment to rehearse before making an actual report relating to an incident, near miss, or CQI.	Yes		✓*	
	Provide a new user with video tutorials on how to report a medication incident or near miss.	Yes		✓	

^a^I or U: important or useful.

^b^Currently implemented.

^c^Not currently implemented.

^d^CQI: continuous quality improvement.

^e^Partially implemented and could be improved.

**Table 3:** Guidelines for incorporating dialogue support principles into Medication Incident Reporting and Learning Systems.

Strategy and implementation			I or U^a^		Status
**Praise**					
	As a show of appreciation, praise or congratulate the user for submitting a near-miss or incident or CQI^b^ report or for reaching a milestone using textual, visual, or audio-based feedback messages [67].	Yes		X^c^	
**Reward**					
	Reward the user with points, badges, etc, when they submit a report (early), achieve a goal or milestone, or others find their report useful (eg, by liking it), etc.	Yes		X	
	Allow the user over time to grow in the value of their contribution to the community. This can be based on the number, frequency, quality, earliness, and usefulness of their reports (to others), for example, from a silver to a gold valuable contributor of the community.	Yes		X	
	Reward the user for reporting or sharing action plans that improved safety in the pharmacy.	Yes		X	
	Reward the user for reporting positive experiences that led to improved safety in the pharmacy, for example, “good news” stories in addition to the negative “error” reports.	Yes		X	
**Suggestion**					
	Suggest to the user from time to time based on their profile, role, or interaction with the system new reports that may be interesting and beneficial to their practice [65].	Yes		X	
	Suggest to the user ways, processes, or methods through which others in the community prevent or address recurrence of certain near misses and incidents.	Yes		X	
	Suggest to the user standard, process-based solutions (eg, from the user’s pharmacy, province, or professional organization) for addressing certain types of recurring incidents and near misses [65].	Yes		X	
	Provide the user with a list of “high-alert” medications or types of incidents that occur most often or require extra precautions and suggest best practices to reduce incidents and near misses associated with them [68].	Yes		✓*^d^	
**Feedback^e^**					
	Provide the user with summary feedback on their progress toward reaching their monthly, quarterly, or yearly goal (eg, “You have achieved 30% of your goal”).	Yes		X	
	Provide the user with summary feedback on the usefulness of their reports to others (eg, “5% of the system users in the province [nation] found your report helpful”).	Yes		X	
	Provide the user monthly, quarterly, or yearly summary feedback highlighting the most recurring types of near misses and incidents (eg, “Poor drug naming caused 5% of the near misses last year”) [65].	Yes		✓^f^	
**Reminder**					
	Remind the user from time to time (eg, based on self-set goals) about the need to report near misses and incidents and about the benefits to other users and patient safety.	Yes		X	
	Remind the user from time to time to complete their CQI action plan that they have started.	Yes		X	
**Verbal persuasion^e^**					
	Allow management such as pharmacy managers and supervisors through personally sent messages to encourage users from time to time to report near misses and incidents, for example, “Alice, remember to report your near misses and incidents to improve patient safety. Yes, you can*!*”	N/A^g^		N/A	
**Emotional appeal^e^**					
	Use motivational messages to encourage users to report errors, for example, “To err is human, to share is divine” [69].	Yes		X	
**Liking**					
	Make the system to be visually attractive, for example, by using visually pleasing or appropriate colors to present charts, content, and important information.	Yes		✓*	

^a^I or U: important or useful.

^b^CQI: continuous quality improvement.

^c^Not currently implemented.

^d^Partially implemented and could be improved.

^e^Not originally listed in the Persuasive System Design taxonomy.

^f^Currently implemented.

^g^N/A: not applicable.

**Table 4:** Guidelines for incorporating social support principles into Medication Incident Reporting and Learning Systems.

Strategy and implementation			I or U^a^		Status
**Social learning**					
	Notify the user by email when other anonymous users submit an incident report (eg, containing the key points) or CQI^b^ report that may be of interest to the user, just as in ResearchGate, for example, “John [a pseudonym], here’s a new report we think you’ll be interested in.”	Yes		X^c^	
	Support chat room and discussion room to foster social support and shared learning [47]. This room can be anonymous.	Yes		X	
	Support a newsfeed (eg, as in Facebook) to highlight important reports the user may find useful and foster shared learning.	Yes		X	
**Social comparison**					
	Allow the user to compare their weekly, monthly, quarterly, or yearly reports with others, maintaining confidentiality (eg, at the city, zone, provincial, or national level).	Yes		✓*^d^	
**Competition**					
	Allow the user to see where they are compared with other anonymous (eg, on a leaderboard) at the pharmacy, provincial, or national level based on the total number, frequency, quality, or usefulness of their report to others (eg, over a weekly, monthly, or yearly period).	Yes		X	
**Cooperation**					
	Provide users the choice of being paired with another anonymous user, with the goal of motivating one another to achieve individual or collective goals.	No		X	
**Normative influence**					
	Inform users about the number of other anonymous users in the pharmacy, province, or nation that are reporting errors in a given period (eg, “10 other people submitted their incident reports today”).	Yes		X	
**Social facilitation**					
	Make users, who are logged onto the system know that there are other anonymous users elsewhere (eg, in the facility, province, and nation), who are submitting or just submitted a report (eg, “5 other people are currently submitting their incident reports”).	Yes		X	
**Social recognition**					
	Provide a means for committed user to be publicly recognized for being one of the “most valuable players” of the month, quarter, or year at the pharmacy, provincial, or national level based on certain criteria (eg, number, frequency, quality, or usefulness of their reports to the community).	Yes		X	
	Allow other users to rate users’ reports anonymously based on how useful or helpful it is to them.	No		X	

^a^I or U: important or useful.

^b^CQI: continuous quality improvement.

^c^Not currently implemented.

^d^Partially implemented and could be improved.

**Table 5:** Guidelines for incorporating system credibility support principles into Medication Incident Reporting and Learning Systems.

Strategy	Implementation	I or U^a^	Status
Authority	Present authority-based information and messages (eg, on the value of reporting incidents and near misses and the benefits it can have for the profession, staff, or patient safety) [47].	Yes	X^b^
Third-party endorsement	Demonstrate that the system is approved by authorities such as professional organizations, regulatory bodies, and government, for example, by displaying their corporate logos [65].	Yes	X
Expertise	The visual and functional design of the system should reflect professionalism, expertise, and be up to date to motivate users to use it.	Yes	✓*^c^
Trustworthiness	Build trust into the system, for example, by fostering anonymity, data aggregation, and keeping promises such as it not being used as a punitive tool to hold users accountable [65].	Yes	✓*
Surface credibility	Build surface credibility into the system through its visual design, for example, by reducing advertisements and ensuring users enter accurate information using taxonomy-based predefined options, checklists, and drop-downs [47].	Yes	✓^d^
Verifiability	Ensure presented information and messages (eg, on the value of error reporting to the profession, staff, or patient safety) are verifiable, for example, through a link to authority-based websites such as Institute for Safe Medication Practices and World Health Organization.	Yes	X
Real-world feel	The design of the system should mimic the paper-based error reporting forms (eg, [70]) as closely as possible to reduce the cognitive effort required by a new user to make the transition [71].	Yes	✓

^a^I or U: important or useful.

^b^Not currently implemented.

^c^Partially implemented and could be improved.

^d^Currently implemented.

Primary support strategies facilitate the key behaviors promoted by the system, such as reporting. Dialogue support strategies enable users to interact, engage with, and receive feedback from the system through text-, image-, audio-, and video-based dialogue. Social support strategies motivate users through social influence. Finally, credibility support strategies enable users to trust and rely on the system. In summary, based on the assessors’ responses, most of the persuasive strategies (29/31, 94%) in the extended PSD taxonomy were considered important or useful, with approximately one-third (14/29, 48%) of them identified as present in the current Think Research or Pharmapod system. Approximately 23% (7/31) and 26% (8/31) of the strategies were considered fully or partially implemented (although they could be improved), respectively. More than 50% (16/31) of the strategies were considered not implemented, with most of them falling under the social support category.

## Discussion

We have presented the results of 2 scoping reviews and the initial assessment of the Think Research or Pharmapod system. The following sections discuss the results with a focus on the persuasive design guidelines shown in Tables 2-5, which can inform the persuasive design of future MIRLS.

### Summary of Scoping Review Findings

Table 1 shows the types of harm uncovered in the first scoping review. More than half (11/17, 65%) of the included articles reported that medicated errors caused serious harm to patients. In particular, 60% (3/5) of the articles reported serious harm, and 40% (2/5) of the articles reported fatal harm or death caused by medication errors such as wrong dose, drug, patient, and ambulatory pump (eg, [58]). Prescription error [16,18,59,63], wrong drugs [12,58,63], and dosing error [58,59,63] were the most frequent medication errors. For example, in the study by Fyhr and Akselsson [58], most severe medication errors occurred during prescribing and transcribing by physicians. The findings are an indication that medication errors have the potential to cause serious harm to patients, including death; hence, there is a need for interventions aimed to reduce them and increase patient safety (eg, by increasing reporting and shared learning within and across organizations). Moreover, in the second review on the usability of MIRLS, George et al [13] found that an iterative design has the potential to improve the usability of an MIRLS. However, their study suggested that there is a need to address issues surrounding data security and report validation to increase user acceptance and use.

### Summary of Administrative and Usability and Utility Assessment

Our assessment shows that the Think Research or Pharmapod system implemented most of the administrative, usability and utility-based principles shown in Textboxes 1, 3 [7,14], and 4 [7,14]. Prior studies advocate most of these principles as essential actions and capabilities aimed at improving incident reporting and shared learning [7,9,14,33-36]. An anonymous reporting, for example, can mitigate the punitive perceptions of incident reporting [20]. However, the system only partially supported persuasive design principles. Persuasive design principles are intended to complement the administrative, usability and utility-based principles by improving the UX and motivating users to see value in reporting MIs and completing the CQI and learning tool reports. Persuasive design may in turn mitigate some of the persistent barriers identified in Textbox 1.

**Administrative guiding principles for designing Medication Incident Reporting and Learning Systems [7,14].**

**Voluntariness**

- Medication reporting will be voluntary.

**Inclusiveness**

- Professionals and consumers will be encouraged to participate.

**Aggregation**

- The reporting system will support anonymity and aggregation.

**Confidentiality**

- The system will provide confidentiality of reported information.

**No consequence**

- The system will clearly define and support a nonpunitive approach to reporting.

**Type of report**

- The system will encourage reporting of both potential and actual incidents and near misses.

**Feedback**

- The system will provide feedback on incident analysis and timely recommendations.

**Workflow alignment**

- The system should fit with the users’ workflow.

**Usability and utility-based guiding principles for designing Medication Incident Reporting and Learning Systems [7,14].**

**Usability**

- The system will be easy to use and time efficient.

**Format multiplicity**

- The system will support both electronic and paper formats.

**Taxonomy**

- The system will support standard taxonomy.

**Outcome severity**

- The system will support levels of severity of outcomes.

**Searchability and retrievability**

- The system will support searchable and retrievable data.

**Report generation**

- The system will support report generation.

**Root cause analysis**

- The system will support root cause analysis.

### Summary of Persuasive Design Assessment

In this section, we discuss the results of the system assessment and persuasive design guidelines for designing future MIRLS, taking each category of the PSD taxonomy at a time.

#### Primary Support Assessment and Guidelines

In the primary support category (Table 2), all the persuasive strategies were considered important or useful, whereas over 55% (5/9) of them were deemed partially or fully implemented by the system.

##### Reduction

Reduction, which is considered important and present in the system by the assessors, entails breaking down the performance of a complex behavior into a few steps. In the context of MI reporting, this means making the reporting process simple and easy to carry out by users. Reduction is vital to ensuring and facilitating the report of MIs and near misses given the relatively high workload health professionals such as pharmacists handle on a daily basis [65]. In the Think Research or Pharmapod system, for example, to speed up the reporting process, predefined fields and system design widgets such as drop-downs are used to enter information about prescribed drugs, what happened, contributing factors, and harm caused. A critical aspect in realizing the effectiveness of the implementation of this and other PSD guidelines is the fit of the MI reporting task into users’ workflow to facilitate regular reporting [7]. However, this examination is beyond the scope of this conceptual study.

##### Tunneling

Similar to reduction, tunneling (aka guided persuasion) aims at motivating users to report MIs and near misses. The tunneling strategy, which can be likened to the process of installing software on a computer using an installation wizard [72], is used to walk the user through predetermined steps in a structured manner. Two of the assessors agreed that tunneling is important or useful but not present in the studied system, with 1 of them remarking, “the report has four sections, then the CQI has colour coded features but they do not tunnel you in any direction.” Once an incident report is completed and saved, the incident analysis interface (an event summary page containing a variety of management tools to prevent the recurrence of similar events in the future) opens automatically. However, the system does not tunnel the user in a specific direction. The third assessor, however, did not find tunneling useful in this context and commented, “No this MIRLS is not like an installation wizard. We like the flexibility provided today.” Hence, owing to the mixed responses, a more comprehensive study among a larger target audience is required to understand the perceived usefulness of tunneling.

##### Goal Setting

Related to the commitment principle proposed by Cialdini [73], goal setting is known as one of the cornerstones of persuasive systems [74]. According to the commitment principle, people are more likely to follow through with a behavior if they make a commitment in written or verbal form to perform the behavior [75]. Studies have shown that people, regardless of culture, are more motivated by the commitment principle than by the other 5 principles of persuasion proposed by Cialdini [76,77]. Goal setting is more likely to be effective if set goals are specific, measurable, achievable, relevant, and timebound (SMART) [77]. The assessors agreed that goal setting is important or useful in MI reporting. One participant thought that the feature was present in the system already. Here, the assessor meant the CQI action planning. In general, both goal setting and action planning are related. However, action planning is concerned with how set goals can be achieved [78]. In the studied system, the CQI actions tool captures both actions (which can be regarded as CQI goals) and action plans (eg, addressing gaps in workflows and processes) [64]. Although we can submit the system-supported CQI goals, it did not support incident reporting goals. Regarding the former, one of the assessors stated that the action plan tab in the system allows a free-form type (such as textboxes that allow the user to type in anything without restrictions). However, it “could be improved by adding prompts for SMART [plans] to guide the user to complete [them] correctly. These action plans are incident specific. They do not allow overall SMART goals around frequency and quality of reporting. [Although], [t]here are dashboards of measurements, they do not include goals or thresholds as a comparison or guide.”

##### Self-Monitoring

Self-monitoring goes hand in hand with goal setting in most implementations [78,79]. In other words, users should be able to visualize their progress toward the realization of their set goals. Self-monitoring is one of the cornerstones of persuasive systems [78] and one of the most requested persuasive features in health apps such as fitness apps [45]. In a systematic review, Matthews et al [80] found that 70% of the included articles evaluated physical activity apps that supported self-monitoring as a persuasive feature to motivate behavior change. Self-monitoring fosters self-reflection and raises users’ consciousness of their responsibilities, which culminates in self-regulation and behavior change [78,81,82]. Self-monitoring can be compared with holding a mirror up to the user’s face, and if the user does not like what they see, they do something about it. In work environments, employees’ engagement in self-monitoring is considered a prerequisite for professional development [82]. In the studied system, self-monitoring is implemented in the form of incident and near-miss reports at the pharmacy, province, or national level. In the data warehouse interface, users can view the number of cases (incidents and near misses); number of events by harm levels, top 5 drugs; and what, why, and when they happened. However, because there is no goal setting for incident report, the system does not support the type of self-monitoring that allows the user to track their progress after setting a report-based goal or when submitting a report, for example, through the display of a progress bar. In addition, the system does not allow the user to track the levels of usefulness of their reports (eg, incident, near miss, and CQI plans) to others. For example, it does not allow other users or colleagues to “like” the user’s anonymous reports or to indicate their usefulness.

##### Tailoring, Personalization, and Customization

All 3 persuasive strategies are related and can be defined as the act of tailoring the user interface elements and content of a system to suit the user’s needs, preferences, designation, or role. Tailoring and personalization are carried out by the system, whereas customization is carried out by the user. Although tailoring is enacted by the system based on users’ predetermined information (eg, gathered through surveys before using the system), personalization is enacted by the system using information gathered in real time (ie, during user interaction with the system) [81,83]. We observed that tailoring was implemented in the assessed system. This system provides role-based access to certain features. However, the assessors remarked that the tailoring feature can be improved depending on what users need. However, we found that the system does not support personalization. Hence, we recommend MIRLS be personalized based on user interaction, for example, letting the user know where they left off or reminding them about incomplete tasks when they log in [7,66]. In addition, we recommend that users be allowed to customize the system (eg, user profile, chart, content, information, and reminder) to suit their needs and preferences [66].

##### Simulation

Simulation is a persuasive strategy used to demonstrate the cause and effect of a given behavior. Although the assessors considered it important, it was not currently implemented in the system. Thus, we recommend that MIRLS provide a means for the user to observe a link between the cause and effect of incident and near-miss reporting [80,84]. A typical implementation of the strategy is demonstrating to the user using a graph or chart that the higher the MIs reported using the system, the lower the number of recurrences.

##### Rehearsal

Rehearsal is a trial performance or practice of a given task so that the user can perform it correctly and easily later. In the assessment, we found that the system already provides a new user with video tutorials (organized in modules) on how to report a MI or near miss. In addition, we recommend that MIRLS provide a new user with a practice environment, in which they can rehearse before using the system to make an actual report.

#### Dialogue Support Assessment and Guidelines

In the dialogue support category (Table 3), all the persuasive strategies were considered important or useful; however, only 50% (4/8) of them (eg, reminder, feedback, and suggestion) were considered partially implemented by the system.

##### Praise and Reward

They entail acknowledging, appreciating, and recognizing the user for their effort and time taken to report incidents and near misses for the benefits of other pharmacists and patient safety. As Holden et al [6] noted, “reward and punishment structures may affect individual reporting decisions (e.g. if nurses are rewarded more for productivity than for reporting), as may culture (e.g. blame vs. just culture).” It is yet to be seen how the web-based rewards implemented in a system may influence error reporting. Enacted through well-worded motivational text and well-designed motivational images, symbols, and sounds [67], praise fosters an intimate relationship between the user and the system, making the user feel valued, appreciated, and more open to persuasion [85]. Although considered important by 2 of the assessors, 1 of them had some reservation. The participant stated, “This would emphatically not be wanted. Reward messages coming from an MIRLS technology should not emulate a sports watch. As an advanced user of the system I would find this annoying and a waste of time. If the system helps reduce incidents, a trend report shows proof, that is praise enough.” However, praise and rewards can be targeted to aggregated reports (eg, a pharmacy) on the basis of the number of incidents that reached and did not reach the patient.

##### Suggestion

This strategy is considered important and partially implemented in the system and can be used as a means of informing users about certain important reports (especially from other anonymous users or generated from the system), which may be useful to them in their practice. A typical suggestion in this context could be a list of “actions to take” for a specific MI or a list of “high-alert” medications that require extra precautions. Other suggestions include new research reports that may be interesting and beneficial to the user or ways, processes, or methods through which other anonymous users in the community prevent or address recurrence of certain medication errors [65]. For example, upon completing a report, the user can be recommended a set of preventive guidelines by the system to mitigate future incidents.

##### Feedback

Several behavior change theories such as social cognitive theory, goal setting theory [86], and feedback intervention theory consider the provision of feedback as an important ingredient in behavior change [87,88]. An example implementation of the self-monitoring-type of feedback is providing the user with summary feedback on their progress toward reaching their goal (eg, “You have achieved 30% of your goal”). Moreover, feedback entails information about one’s behavior or system-generated figures and statistics. In the context of MIRLS, informational feedback is the information of the user about the impact of their error-reporting behavior on the community or health providers’ medication errors on patient safety. An example of informational feedback is informing pharmacists about the usefulness of their reports to other users in the community (eg, “5% of the system users in the province [nation] found your report helpful”). Another example is providing users with monthly, quarterly, or yearly summary feedback highlighting the most recurring types of errors relevant to their work [65] (eg, “Poor drug naming caused 5% of the near misses last year.” In addition, the solution to this medication error can be included in the feedback message as well; for example, “Poor drug naming caused 5% of the near misses last year; remember to use TALLman lettering when necessary.” The use of uppercase letters in a portion of a drug name helps to draw attention to the dissimilarities between look-alike and sound-alike drug names. Moreover, it helps to alert health care professionals that the name of a given drug can be confused with another drug that has a similar name [89].

##### Reminder

This refers to an alert on task completion and compliance with certain behavior or expectation [90]. Reminder is closely tied to goal setting in a certain regard. For example, if the user sets a goal (eg, report at least X errors per month), then the user should have the opportunity to set reminders so that they could be reminded at certain preset times to report incidents or near misses if they have any. Reminder has been widely and successfully used in persuasive systems, especially in the health domain, to motivate behavior change [80,91]. In MIRLS, reminders, considered important and partially implemented, can be based on users’ self-set goals on medication error reporting as well as CQI-based action plans. For example, based on self-set goals, the system can remind the user at preset times about the need to report near misses and incidents when they occur and about the benefits of the reports to other users in the community and patient safety. For instance, the system can prompt the user at a preset time with a message such as, “Did you have any near misses today or in the last one week? Please report if you did.” Moreover, the system can remind the user through this type of message if the user has not logged into it or submitted a report within a certain period. In addition to this reminder-based messages, a direct link to a reporting wizard can be included, allowing users to easily submit a report by simply clicking on the provided link. Persuasive reminders have been widely used in health self-management such as taking one’s daily medication and have been effective [92]. Although reminders may be more effective if they are just-in-time [87], in the context of MIRLS, they can be well ahead of time, for example, during the period when a user such as a pharmacist resumes their shift. They can also be at the end of the pharmacist’s shift. Therefore, research, in the context of MIRLS, is required to show which of the periods (start or end) is more likely to be effective in motivating reporting of medication errors. In summary, reminders can be general or specific. General reminders are aimed to remind users from time to time to report incidents if they have any. Moreover, specific reminders are aimed to remind users to complete incident report drafts (ie, reports that they started but have not completed). Nevertheless, reminders should be used with caution as they can be overwhelming if overused. As stated by 1 of the assessors, “Reminders can also be annoying to the point of reminder fatigue and disregarded instantly, and overkill for this type of solution.” Therefore, users should be allowed to turn them on and off.

##### Verbal Persuasion

This refers to the act of mentoring and providing encouragement and feedback to help individuals achieve their goals. It is also defined as “the act of telling or convincing a person to perform a task or action to change a behavior or put into action a set of events to achieve an objective” [93]. Research shows that organizational and leadership coaches use verbal persuasion effectively to increase the self-efficacy of their clients and the results they create. The tools for carrying out verbal persuasion include praise (kind words about the user), encouragement (words of affirmation about the user’s ability), stories (personal or allegorical stories to help reframe the user’s struggle with the task), positive feedback (assessing the user’s performance favorably), strengths focus (intentionally linking the task to the user’s strengths), and past achievements (acknowledging past wins as an indication of the user’s ability to complete the current task) [94]. In the context of MIRLS, praise and encouragement may be used effectively by community pharmacy managers and supervisors to motivate users to report near misses and incidents. However, the use of individual feedback and past achievements may not be possible in MIRLS if, at the pharmacy level, managers and supervisors do not have access to individual users’ performance owing to anonymity. In the event that managers had access to individual users’ performance, as may be the case in certain pharmacies owing to corporate policy, managers and supervisors could enact verbal persuasion through personal feedback and strengths in addition to praise and encouragement. Although verbal persuasion can be said to be related to the praise and emotional appeal strategies, the main difference is that verbal persuasion is coming directly from a superior (eg, a pharmacy manager) that the user knows rather than the system. A typical message a pharmacy manager can send to an employee to verbally persuade them is, “Alice, remember to report your near misses and incidents to improve patient safety. Yes, you can*!*” Moreover, a typical feedback message from a pharmacy manager is, “Alice, thanks for your constant reporting of near misses—keep it up!” Users (whether reporting frequently or not) may find this type of message motivational. This may motivate users who have not been reporting their errors using the system in recent times to start reporting. Moreover, this type of positive feedback will help address one of the administrative barriers presented in Textbox 1: “Underreporting due to lack of useful feedback or negative feedback from administrative teams such as pharmacy managers” [8].

##### Emotional Appeal

It is a persuasive strategy designed to elicit an emotional response based on feelings [95]. We argue that motivational messages that appeal to emotion and feeling, such as “To err is human, to share is divine” [69], have the potential to motivate users in the medication error–reporting domain, similar to other domains [81]. In the fitness app domain, for example, Oyibo [96] found that, regardless of gender, health messages that appeal to emotion, such as *“*Those who do not find time for exercise will have to find time for illness,” have the potential to motivate people to start or continue exercising. However, in this study, we found that although a motivational message such as “To err is human, to share is divine” may motivate some pharmacists, as evident in 1 of the assessors’ responses (“would love it”), it may demotivate others. One of the assessors commented that the use of emotional appeal is inappropriate in a professional domain such as community pharmacy. The assessor stated, “It is a regulatory requirement to report incidents–no need for motivational messages...like a sports watch or fitbit. It seems unprofessional for a tool such as this to have this. I would NEVER accept this or turn this feature on.” The mixed reactions to the use of emotional appeal to motivate incident reporting, similar to praise and reward, require further empirical studies.

##### Liking

This entails making a system visually attractive and engaging to make it persuasive. This strategy in the PSD taxonomy is drawn from the 6 principles of persuasion proposed by Cialdini [73]. According to Cialdini [73], the more people like someone, the more likely they are to be persuaded by the person. Similarly, in the context of PSD, the more esthetic a system is, the more persuasive users find it and the more likely the users are willing to use it to motivate their behavior change [48,97]. In the context of MIRLS, designers can use visually pleasing user interfaces and appropriate colors to present charts, content, and important information to improve the overall UX.

#### Social Support Assessment and Guidelines

##### Overview

In the social support category (Table 3), we only found that social comparison (in the form of benchmarking) was already implemented in the system for a limited number of measurements. However, the user had to filter each time to be able to benchmark the measure of interest (eg, near miss) at one level (eg, in the pharmacy) against another (eg, in the province). The assessors of the system suggested that rather than filtering all the time, it would be better if the benchmarking feature of the system could be enhanced by locking in the error reports—having them appear automatically. Moreover, we recommend guidelines on how to integrate other socially oriented persuasive strategies such as social learning, social facilitation, normative influence, competition, and social recognition. Holden and Karsh [7] found that social influence at the individual, group, organizational, and industry levels has the potential to influence medication error reporting.

##### Social Learning

This social strategy allows users to observe and imitate the behaviors and achievements of other (anonymous) users of the system [98]. The social learning strategy derives from the social learning theory proposed by Bandura [99]. The social learning theory states that people have the ability to imitate new behavior by coding or storing the ideas about the behavior in their memory, which eventually guide the actual performance of the behavior [100]. In the context of persuasive technology, social learning is simply implemented using the information of the target user about a target behavior performed by other users, for example, through a notification. In the context of MIRLS, a potential approach to implementing social learning is by enabling users to receive notifications (eg, via email) when fellow users in their group submit incident reports. These notifications would contain essential key points from the submitted reports. A typical notification message to this effect is “John [a pseudonym], here’s a new report we think you’ll be interested in.” We believe that messages such as this, which enable one user to learn from others’ reports, may motivate the target user to submit their reports given the benefit they derive from them. Given that users may be overwhelmed, they should be given the opportunity to determine the types of messages they wish to receive, the number within a given period such as a week or month, and even opt out completely by turning the feature off. More importantly, owing to privacy concerns, particularly within a facility setting, instead of basing the social learning strategy on key points from reported near misses or incidents, it can be based on the quantity of reports submitted within a specified period (refer to the *Normative Influence* section). According to 1 of the assessors, “I don’t think this [first Social Learning implementation] is appropriate if you can see who it is but if it is just numbers it would be useful. [N]otification within a facility could hamper the feeling of safe reporting because anonymity is compromised.” A second implementation of social learning is the provision of a news feed that highlights important reports submitted by other anonymous users that the user may find useful. A third implementation is the support of chat rooms or discussion rooms where users can discuss near misses, incidents and lessons learned; share experiences and knowledge; and learn from one another in an anonymous fashion. The chat room and discussion forum feature may be extended and beneficial to nonpharmacists, as evident in 1 of the assessors’ comments, “Our users may find this useful. If they have the time, which currently they don’t have much of during the pandemic.”

##### Social Comparison

Social comparison allows users to compare their performance with that of others. It is derived from the social comparison theory proposed by Festinger [101], which centers on the belief that individuals have an inner drive to gain accurate self-evaluations through social comparison. It holds that by comparing one’s abilities and performances with those of similar others or peers, the individual is able to reduce uncertainty, learn, and improve self. This strategy has been used successfully in persuasive systems [102]. In the assessment of the Think Research or Pharmapod system, we found that social comparison was implemented at the pharmacy and provincial level in the form of benchmarking reports, tables, and dashboards. For example, 1 of the assessors responded thus, “within our own organization we may compare pharmacies with other pharmacies or between provinces of our pharmacies using reports provided.” Thus, the implementation of social comparison in the system can be improved. For example, users’ error reporting over a particular period, for example, week, month, or year, can be compared anonymously with the average at the pharmacy or provincial level using a bullet chart infographic.

##### Competition

Similar to social comparison, competition allows users to compare themselves with others, for example, in terms of number of reports, frequency, quality, or usefulness of reports to others. Competition leverages the natural drive of humans to outperform one another [98]. Research on persuasive technology shows that competition, regardless of gender, age, and culture, has the potential to motivate users to perform the target behavior [103]. In the fitness app domain, for example, Oyibo and Vassileva [98] found a significant relationship among social comparison, social learning, and competition, indicating that the more people compare themselves, the more they learn about the performance or achievements of others and the more competitive they become in their behaviors. In the context of MIRLS, users can be allowed to view where they are compared with other anonymous users in small sets (eg, on a leaderboard). The criterion for placement on the leaderboard can include the total number of reports, frequency, quality, or usefulness of the report to others (eg, over a weekly, monthly, or yearly period). The small sets of anonymous users can be drawn from the pool of users at the provincial or national level, which can change from time to time because of the need to foster anonymity. Moreover, the competition feature can be group based, involving anonymous pharmacies, organizations, or provinces. As 1 of the assessors remarked, “Perhaps [my organization] may wish to see how many incidents they are experiencing compared to another organization of the same industry channel and size.”

##### Cooperation

Unlike competition, where users compete to outperform one another, in cooperation, users work together in a collaborative fashion to achieve their individual and collective goals. In the assessment of the Think Research or Pharmapod IM system, we found that providing users the choice of being paired with another (anonymous) user, with the goal of motivating one another to achieve individual or collective goals may not be a good idea. This is based on the premise that the implementation of cooperation in MIRLS may compromise the principle of anonymity of users, upon which MI reporting is founded. Hence, we recommend that cooperation be implemented and used with caution if MIRLS were to support it in a given pharmacy. As commented by 1 of the assessors, “Why would anyone wish to be compared to [cooperate with] another user? Where’s the privacy aspect of such a feature?”

##### Normative Influence

Unlike informational influence, which is conformity to a certain behavior based on the acceptance of evidence about reality provided by others, normative influence is conformity based on an individual’s desire to fulfill others’ expectations to gain acceptance, fit in, or feel a sense of belonging [104]. In the context of reporting medication errors, the urge for individual users to report near misses and incidents might arise from perceived social pressure rather than actual pressure, considering that the submitted reports are anonymous or deidentified. Thus, a possible way of realizing the normative influence strategy in MIRLS is allowing the user to know about the number of other anonymous users in the facility, province, or nation that are reporting medication errors at a given time. For example, in COVID-19 contact tracing apps, Oyibo and Morita [105] found that socially oriented messages, such as “112 other people reported their COVID-19 diagnosis today,” have the potential to motivate app users to report their diagnosis by entering their one-time key into the app. Hence, we recommend that the system informs users at suitable intervals (eg, when they are logged on) about the quantity of other anonymous users within the pharmacy, province, or country who are reporting medication errors within a specific period. A message similar to the message by Oyibo and Morita [105], “10 other people submitted their incident reports today,” may be used to normatively influence users to submit their own incident reports as well if they have any pending or have not yet submitted.

##### Social Facilitation

Social facilitation refers to the improvement in a person’s performance as a result of the real, imagined, or implied presence of others. As stated in the study by Mohadis et al [84], “System users are more likely to perform a targeted [behavior] if they discern, via the system, that others are performing the [behavior] along with them.” In MIRLS, one way to realize social facilitation is to inform the user when they log on to the system (eg, to make a report) through news feed that they are not alone in their efforts to report an error, as other users elsewhere (eg, in the facility, province, or nation) at the current time are also attempting to making a report or logged on to the system. Motivational messages such as “You are not alone; X others are on the system at the moment submitting a report” could be used to make the user feel the presence of other anonymous users whenever the former is logged into the system. A message such as this may encourage users, who have begun the process of submitting a report, to complete it. This type of message is similar to that which customers get when they are booking a hotel or shopping for a flight ticket on the web (eg, “5 other people are currently shopping for this flight ticket”). Although this type of message is commonly used in the e-commerce domain to create the impression that the user may miss procuring a given flight ticket if they do not act quickly (ie, buy it now), in the domain of medication error reporting, this is not the case. Rather, this type of message is used to let the user know that they are not alone—that there are similar others elsewhere who are trying to do the same task as them (submit a MI report).

##### Social Recognition

In social psychology, social recognition is the act of recognizing people such as employees for great work, contribution, and achievement by acknowledging them publicly. One possible way of implementing this strategy in an MIRLS is recognizing users for being one of the “most valuable players” of the month, quarter, or year. This can be at the facility, provincial, or national level. The criteria for recognition include the number, frequency, quality, or usefulness of the target user’s reports to the community. Although research shows that employees welcome social recognition in the workplace [84], it must be implemented with caution given the anonymity requirement aimed to protect users from punitive measures. We found that users may not welcome the second feature (“allowing other users to rate a user’s report anonymously based on how useful or helpful it is to them”) as they perceived it as a form of competition. For example, 1 of the assessors commented, “Rating makes this feel like a competition or to call out that can produce negative attitudes. Not helpful. Those entering data into a system may not be the same person who is involved in the incident.” Moreover, the user was also concerned about the part of the report being rated as well as privacy and anonymity, “What part of the report is being rated in this scenario?” It is worth noting that we conceived the social recognition rating feature similar to Google Play Store app rating system, in which users can rate an app on a 5-star scale. Although we did not explicitly detail the section of the report being anonymously rated by other users in the study, we intended it to encompass essential elements derived from the report analysis, such as the description of the near miss or incident, the lessons learned by the reporters, and possible recommendations and tips to prevent future recurrence. These key points may have been extracted from a set of similar aggregated reports submitted by different anonymous users at different times and included in the MI analysis report shared with users via the MIRLS by standard bodies such as Assurance and Improvement in Medication Safety (AIMS) [106]. AIMS is a standardized medication safety program that supports CQIs and sets a mandatory consistent standard for medication safety for all pharmacies in Ontario. Its goal is to minimize the risk of harm to patients caused by MIs in the province. Part of its mandate is to aggregate and analyze anonymous MI reports and produce and disseminate the results to stakeholders. This enables practitioners to learn from MIs and have a better understanding of why they occur and how they can be prevented in the future [106]. Although in this study, we did not find the second social recognition feature to be useful to the assessors, there may be a need for a more comprehensive study in future research among a larger audience of community pharmacists to uncover its potential to motivate users to report medication errors more frequently.

#### System Credibility Support Assessment and Guidelines

Regarding the credibility support category (Table 4), the assessors reported that the system fully or partially supported a number of credibility-related persuasive strategies such as trustworthiness, credibility, expertise, and real-world feel. We discuss all these strategies together with the other 3 strategies in the credibility support category.

##### Authority

One of the principles of persuasion proposed by Cialdini [73], the authority principle, states that people are more likely to believe and obey those who are in positions of authority. Selassie et al [76] found that frontline staff working with children with autism (supported by a data entry management system) can be persuaded by the authority strategy. Moreover, in the study by Mohadis and Ali [84] on user perception of a physical activity app for older workers, 1 of the participants remarked, “Yeah, incorporating an expert [authority figure’s] view is very important so that we become more confident with whatever recommendations that the system offers.” In the context of community pharmacy, authority figures and bodies may include researchers, pharmacy managers, and professional bodies such as the Institute for Safe Medication Practices Canada [1]. Thus, we recommend the presentation of authority-based information and messages to users, for example, on the value of reporting medication errors and the benefits it can have for the profession, staff, and patient safety [47].

##### Third-Party Endorsement

Third-party endorsement is the act of publicly approving or supporting a product, system, or service by a reputable socially influential individual or organization other than the staff or company that owns it. Usually, the third party may have seen, interacted, and used the product, system, or service in question and is satisfied with the results, utility, or experience. In the business world, research has shown that the third-party endorsements have the potential to effectively earn companies the trust and loyalty of customers [21,69]. Moreover, research shows that the expertise and trustworthiness of a third-party organization endorsement have the potential to positively affect the perceived value of a firm, which in turn can positively affect customer loyalty [107]. Hence, to encourage pharmacists to use MIRLS, the designers should demonstrate that the system is approved or endorsed by authoritative bodies such as professional organizations (eg, World Health Organization and Institute for Safe Medication Practices), regulatory bodies, and government. To implement this persuasive strategy in MIRLS, one approach is to incorporate the corporate logos of the endorsing authoritative bodies within the user interface, such as on the system’s home page or in the footer, especially if it is a web-based application.

##### Expertise, Surface Credibility, and Trustworthiness

Research has shown that all 3 strategies are related. For example, Fogg and Tseng [108] postulated that credibility, a perceived quality of a system, comprises 2 key components: trustworthiness and expertise. In other words, a system is perceived to be credible if its perceived trustworthiness and perceived expertise are high. Trustworthiness is a key element in the credibility perception of systems such as websites. It is defined by terms such as well intentioned, truthful, and unbiased [109]. As stated in the study by Fogg et al [109], “the trustworthiness dimension of credibility captures the perceived goodness or morality of the source.” Similarly, expertise is a key element in the credibility perception of systems such as websites. It is defined by terms such as knowledgeable, experienced, competent, and professional [109]. As stated in the study by Fogg et al [109], “[t]he expertise dimension of credibility captures the perceived knowledge and skill of the source.” In a large-scale website credibility study conducted by Fogg et al [109], the authors found that perceived expertise and perceived trustworthiness have a significant impact on the perceived credibility of websites. In the context of MIRLS, to realize expertise, the visual and functional design of the system should reflect professionalism, expertise, and up-to-dateness to motivate users to use it. Moreover, to implement trustworthiness, the system should foster user anonymity, data deidentification, and data aggregation and live up to promises such as it not being used as a punitive tool to hold users accountable [65]. Finally, perceived credibility can be intentionally built into the system through its visual design, for example, by ensuring users enter accurate information using taxonomy-based option buttons, checklists, and drop-downs and reducing advertisements for a web-based system [47]. In our study, all 3 assessors agreed that perceived expertise is important or useful as well as implemented to a great extent in the system they were currently using. For example, 1 of the assessors commented, “The MIRLS is very easy to use and intuitive, and requires minimal training to get started.” However, “there is always room for improvement,” remarked another assessor. Failure to foster expertise in the system design may discourage frequent use and completion of tasks, as evident in the assessor’s comment, “Performance in speed is always a challenge and [the] latency [experienced in some] areas drive users to drop off or stop using.” Regarding trustworthiness, 2 assessors considered it important or useful. However, only 1 assessor considered it to be implemented in the current system. This is partly because of anonymity not being completely fostered in the system. This is evident in 1 of the assessors’ comments, “anonymity is fostered outside an organization (eg. when data sent to AIMS) and there is also a choice to report anonymously so the corporate level of an organization does not have visibility. [W]ithin a location the reports are not anonymous.” Finally, regarding surface credibility, 2 assessors considered it important or useful and implemented it in their current system. For example, all 3 assessors responded that there were no advertisements in the system and that was very important.

##### Verifiability

This refers to “the quality or state of being capable of being verified, confirmed, or substantiated” [110]. In the context of MIRLS, persuasive messages (eg, on the value of error reporting to patient safety) aimed at motivating users should not only be credible but also verifiable. As stated in the study by Jones [111], carefully choosing persuasive messages and supporting materials that are verifiable, specific, and unbiased can be helpful in appealing to logic and increasing users’ trust. Verifiability was implemented in WargaFit (a fitness app prototype aimed to encourage simple exercise such as body stretching in an office environment) by the provision of healthy tips accompanied with external links [84]. Similarly, verifiability in MIRLS can be realized through the provision of the source of information or inclusion of the URL in the persuasive message such as “Reporting reduces the number of future errors, diminishing personal suffering and decreasing financial costs” [112]. In our study, 2 assessors considered verifiability useful and not currently implemented in their system. For example, regarding harm levels, 1 of the assessors commented, “There are info points that explain [that] harm level comes from WHO but there is no link to the WHO to verify it.”

##### Real-World Feel

Similar to expertise and trustworthiness, real-world feel is found to positively influence the perceived credibility of websites [109]. Real-world feel is the interaction with and experience of a virtual or electronic product, system, or service as though it is real. This is made possible by the product-, system-, or service-supporting features that mimic and foster real-world interaction and experience. In the case of e-commerce websites, for example, the real-world feel can be fostered by providing contact phone number, contact email address, and a quick response to customer service questions; listing the physical address of the organization behind the website; and showing photos of the members of the organization [109]. In the context of MIRLS, in addition to the aforementioned features, the system should be designed as close as possible to the nonelectronic (paper) version. This has the potential to reduce the cognitive effort required by a new user to make the transition. In the assessment of the Think Research or Pharmapod IM system, assessors stated that it supports real-world feel by mimicking the paper version and allows clients to customize their own forms and notifications or escalations. One way the system designers achieved real-world feel is to allow pharmacies and organizations to customize their MI report forms.

### Persuasive System Implementation and Ethical Design Considerations

Our analysis reveals that there is a need to consider and address the ethical implications that may arise from integrating persuasive strategies into the existing MIRLS. These considerations include administrative (eg, anonymity) and choice of persuasive strategies (eg, monetary reward). For example, to ensure that the principle of anonymity is fostered in the implementation of social strategies, user identifications should be limited to pseudonyms, which the users can change from time to time. It is worth noting that a persuasive strategy that may be effective (or welcomed) in one community pharmacy may not be in another. Hence, there may be a need to get the potential users involved in deciding the set of persuasive strategies that will be implemented or effective in a given pharmacy. Thus, the system should offer tailoring capabilities that support the chosen guidelines. Intervention researchers and designers may have to (1) investigate, before implementation, which of the recommended persuasive strategies a given group of pharmacy professionals may be or may not be receptive to and (2) implement only the set of strategies that are likely to be effective, as proven by empirical evidence. For these reasons, MIRLS should be designed in a way that enables pharmacies to turn on and off persuasive strategies that they consider useful and nonuseful, respectively. It is worth noting that some of the persuasive strategies in the PSD taxonomy may have to be combined to realize a holistic and functional persuasive feature that is useful. In other words, some of the persuasive strategies are complementary. For example, praise and feedback strategies must be combined to implement or realize a composite feature that provides immediate feedback of praise to the user upon submitting an incident report. In addition, reminders and verbal persuasion may be combined to realize a composite feature that verbally persuades the user through a reminder. For example, a verbal persuasion message (“Alice, remember to report your near misses and incidents to improve patient safety. Yes, you can*!*”) can be sent to the user as a reminder by the pharmacy manager from time to time. Finally, authority, credibility, and verifiability may have to be combined to realize a persuasive message that is not only authoritative and credible but also verifiable.

### Contributions

In this study, we have made a number of contributions to knowledge in the domain of community pharmacy and developers of health digital systems. This study is the first to provide guidelines on how to integrate persuasive strategies into MIRLS to increase their utility and motivate users to report MIs and near misses to improve patient safety and promote shared learning. Specifically, we provided MIRLS-specific persuasive design guidelines based on the PSD taxonomy proposed by Oinas-Kukkonen and Harjumaa [30]. Most of the PSD guidelines in the extant literature are concentrated in the domains of healthy eating [113] and physical activity [81,84,114]. Designers of MIRLS can leverage the current set of PSD guidelines in improving future iterations not only in community pharmacy but also in other settings where incident or error reporting is essential and part of the organizational practice. The second contribution is that this study lays the foundation for future empirical research aimed at investigating the effectiveness of persuasive strategies incorporated into MIRLS. Future research efforts should focus on ≥1 of the design guidelines in each of the 4 categories of the PSD taxonomy; implement them; and conduct a field study to examine the perception, acceptance, and adoption of the implemented strategies by the target community pharmacists.

### Research Directions

In future work, we look forward to investigating the potential effectiveness of some of the proposed persuasive design guidelines presented in Tables 2-4 and Textbox 4 in field studies. First, we will create prototypes of the persuasive strategies and perform an empirical study to explore which set of strategies might be more effective. In addition, we will analyze the potential influence of demographic variables, such as age, gender, and work experience, on the effectiveness of these strategies. Second, we will select the most persuasive strategies that the target community of pharmacy professionals are most responsive to and implement them in an actual MIRLS (eg, Think Research or Pharmapod). Third, we will conduct a field study (randomized controlled trial) to investigate the effect of the persuasive design on the rate of MI reporting among community pharmacy professionals using different provinces across Canada as case studies. More importantly, owing to the lack of studies on the relationship between system usability and medication error reporting, as our second scoping review shows, we recommend that future work be conducted in this area.

### Limitations

Similar to most conceptual papers, our study has limitations owing to its preliminary nature, which stems from the nonmaturity of research on the persuasive design of MIRLS. The first limitation is that the results of the scoping reviews might have been limited one way or the other by the choice of search strings and the subjective assessment, understanding, and interpretations of the extracted data by the researchers that conducted the reviews. Hence, we recommend a more comprehensive review, particularly with regard to the second RQ, in which a formal review led to no included article, other than the article retrieved from Google Scholar search. The second limitation of our study is the convenience sample. In other words, the 3 assessors who assessed the Think Research or Pharmapod system using the PSD taxonomy were not sufficient to be representative of the entire population of community pharmacy professionals using MIRLS across Canada. For example, a persuasive feature that may be important and useful to a group of community pharmacists in one facility may not be useful to another group in another facility. Hence, the findings reported in the last 2 columns of Tables 2-4 and Textbox 4 may not generalize to a larger population sample involving a heterogeneous group of community pharmacists with different roles, working environments, years of working experience, professional qualifications, gender, personality, and economic status, which may influence their responses. In future work, we hope to build on this preliminary study by conducting a formal research (eg, based on storyboards) involving a larger population sample to validate the generalizability of the findings of this study, particularly the effectiveness, acceptability, and adoption of the recommended persuasive strategies presented in Tables 2-4 and Textbox 4.

### Conclusions

Although most medical practitioners agree that reporting medication errors improves the quality of care and safety for patients [21], in reality, the rate of reporting remains below expectations [115] owing to lack of motivation and other barriers [22-25]. In this study, we argued that although most current MIRLS have implemented recommended guidelines bordering on favorable administrative measures and utility, they lack motivational affordances that can facilitate or motivate frequent reporting. Hence, using the Think Research or Pharmapod system as a case study, we identified opportunities for incorporating persuasive strategies into MIRLS to make them more effective in motivating behavior change. The proposed persuasive design guidelines can be used by designers and developers in making MIRLS more effective in motivating users to report incidents and near misses more often to reduce risks of recurrence, improve patient safety, and foster shared learning among community pharmacy professionals and stakeholders. However, before the implementation of the recommended persuasive design guidelines in Tables 2-4 and Textbox 4, there is a need for thorough consideration and evaluation of the various ramifications, including administrative, regulatory, and ethical implications. The presented persuasive design guidelines open up new opportunities for persuasive design research in MI reporting. We acknowledge that some of the proposed persuasive strategies may not be suitable or effective in real-life settings. Hence, there is a need for further validation-based research and caution regarding their implementation. In future work, we aim to validate the suitability and effectiveness of the proposed persuasive strategies in motivating behavior change using storyboards, prototypes, and perception and evaluation studies involving community pharmacists across Canada.

## Abbreviations

AIMS: Assurance and Improvement in Medication Safety
CQI: continuous quality improvement
IM: Incident Management
MI: medication incident
MIRLS: Medication Incident Reporting and Learning Systems
PSD: Persuasive System Design
RQ: research question
SMART: specific, measurable, achievable, relevant, and timebound
UX: user experience
